# Band Gap Opening in Borophene/GaN and Borophene/ZnO Van der Waals Heterostructures Using Axial Deformation: First-Principles Study

**DOI:** 10.3390/ma15248921

**Published:** 2022-12-13

**Authors:** Michael M. Slepchenkov, Dmitry A. Kolosov, Igor S. Nefedov, Olga E. Glukhova

**Affiliations:** Institute of Physics, Saratov State University, Astrakhanskaya street 83, 410012 Saratov, Russia

**Keywords:** van der Waals heterostructures, density functional theory, band structure, density of states, buckled triangular borophene, gallium nitride, zinc oxide, uniaxial and biaxial stretching/compression

## Abstract

One of the topical problems of materials science is the production of van der Waals heterostructures with the desired properties. Borophene is considered to be among the promising 2D materials for the design of van der Waals heterostructures and their application in electronic nanodevices. In this paper, we considered new atomic configurations of van der Waals heterostructures for a potential application in nano- and optoelectronics: (1) a configuration based on buckled triangular borophene and gallium nitride (GaN) 2D monolayers; and (2) a configuration based on buckled triangular borophene and zinc oxide (ZnO) 2D monolayers. The influence of mechanical deformations on the electronic structure of borophene/GaN and borophene/ZnO van der Waals heterostructures are studied using the first-principles calculations based on density functional theory (DFT) within a double zeta plus polarization (DZP) basis set. Four types of deformation are considered: uniaxial (along the Y axis)/biaxial (along the X and Y axes) stretching and uniaxial (along the Y axis)/biaxial (along the X and Y axes) compression. The main objective of this study is to identify the most effective types of deformation from the standpoint of tuning the electronic properties of the material, namely the possibility of opening the energy gap in the band structure. For each case of deformation, the band structure and density of the electronic states (DOS) are calculated. It is found that the borophene/GaN heterostructure is more sensitive to axial compression while the borophene/ZnO heterostructure is more sensitive to axial stretching. The energy gap appears in the band structure of borophene/GaN heterostructure at uniaxial compression by 14% (gap size of 0.028 eV) and at biaxial compression by 4% (gap size of 0.018 eV). The energy gap appears in the band structure of a borophene/ZnO heterostructure at uniaxial stretching by 10% (gap size 0.063 eV) and at biaxial compression by 6% (0.012 eV). It is predicted that similar heterostructures with an emerging energy gap can be used for various nano- and optoelectronic applications, including Schottky barrier photodetectors.

## 1. Introduction

The stacking of atomically thick 2D materials in the form of a vertical layered heterostructure opens up wide opportunities for obtaining new materials with unique properties [1,2]. Since the layers in a vertical heterostructure are bound only by weak van der Waals forces, it becomes possible to combine atomically thin structures with different crystal structures and lattice parameters [3]. To build vertical heterostructures, such 2D materials as graphene [4], boron nitride [5], zinc oxide [6], molybdenum disulfide [7], tungsten diselenide [8], as well as representatives of the MXenes groups [9] and metal-organic compounds (MOFs) [10], are already used. A wide variety of electronic and optical properties of modern 2D materials determines the wide possibilities of using van der Waals heterostructures in various devices, including vertical field-effect transistors, highly sensitive photodetectors, and some magnetoelectric devices [11,12,13].

Promising results in the design of van der Waals heterostructures with the desired properties are expected from borophene. Borophene is an experimentally obtained new 2D material consisting of boron atoms [14,15]. The atomic structure of borophene contains triangular and hexagonal elements. The existence of various allotropic forms in borophene significantly reduces the requirements for matching the crystal lattice of 2D crystals in a vertical heterostructure. Due to such unique properties such as its lightness, mechanical strength and flexibility, high thermal and electrical conductivity, and optical transparency, borophene is considered to be one of the most promising single-element 2D materials for nano- and optoelectronics [16]. The first successes have already been achieved in the field of creating heterostructures based on borophene and studying their properties. X. Liu et al. [17] and C. Hou et al. [18] reported on the synthesis of vertical borophene–graphene heterostructures. Despite the minimal similarity of crystal symmetry and lattice constant between graphene and borophene sheets, graphene/borophene heterostructures have an almost perfect atomic consistency. Based on the results of the environmental humidity measurements, it was found that the borophene–graphene heterostructure demonstrates a high sensitivity (4200%) and fast response/recovery time (10.5 s/8.3 s) with long-term stability and flexibility. This allows it to be used to create a high performance humidity sensor [18]. According to first-principles calculations, borophene/graphene heterostructures can be considered as a promising anode material, exhibiting high Li adsorption energy (−2.959 eV) and high theoretical specific capacity (1469.35 mA h/g) [19].

Computer simulation methods are used to search for new configurations of borophene-based vertical heterostructures, predict their properties, and analyze the possible applications. Using ab initio methods, Kochaev et al. predicted the appearance of a vertical heterostructure based on borophene with a periodic perforation and graphenylene [20]. The researchers showed that the band gap of the borophene-graphenylene heterostructure can be controlled by tensile deformation and the passivation of the carbon layer by atomic hydrogen. The mechanical properties of the borophene-graphenylene heterostructure correspond to the mechanical properties of graphenylene. Vishwakarma et al. reported the discovery of interfacial coupling quantum states in borophene/boron nitride and borophene/MoS_2_ heterostructures [21]. It was predicted that quantum-coupled borophene-based heterostructures can become promising materials for exciton and molecular sensors. The adsorption behavior of CH_4_, C_2_H_4_, C_2_H_2_, CH_3_OH, and HCHO organic molecules on borophene in the borophene/MoS_2_ van der Waals heterostructure was studied by the DFT method [22]. The calculations of the sensory characteristics allowed us to predict that the borophene/MoS_2_ heterostructures can be used as an excellent gas sensor for organic molecules such as C_2_H_2_ and HCHO.

One of the hot research topics in the last few years is the implementation of a metal-semiconductor contact based on borophene heterostructures. In 2020, Katoch et al. performed an ab initio study of the prospects for using borophene in combination with MX_2_ (M = Mo, W and X = S, Se) to form a metal-semiconductor contact. Scientists have shown that deformed β_12_/MX_2_ heterostructures can be used to implement tunable Schottky barriers [23]. The electronic properties of borophene/InSe van der Waals heterostructures have been studied using ab initio methods [24]. It has been found that an ohmic contact can be realized in the borophene/InSe heterostructure by changing the interlayer distance. By applying an external electric field, one can control the height of the Schottky barrier in the borophene/InSe heterostructure. Jiang et al. performed an ab initio study of the electronic properties of borophene/g-C2N van der Waals heterostructures [25]. The results of the study showed that an external electric field can be used to control the concentration of carriers between the borophene and g-C2N interfaces, inducing a transition from an n-type Schottky contact to an ohmic contact. Thus, computer simulation methods and approaches make it possible to find optimal ways to tune the electronic and optoelectronic parameters of borophene-based van der Waals heterostructures. In addition, ab initio methods such as PBE-DFT help to understand the synergy between the mechanical, electronic properties, and experimental problems for different 2D materials [26,27].

In this paper, attention is drawn to the new atomic configurations of borophene-based 2D van der Waals heterostructures, namely borophene/GaN and borophene/ZnO. These configurations include a buckled triangular borophene, which has the highest energy stability among other allotropic forms of borophene [28]. Previously, van der Waals heterostructures based on buckled triangular borophene were not considered. Graphene-like ZnO and GaN monolayers have crystal lattice parameters close to those of borophene, which makes it possible to significantly reduce the number of atoms in the calculated supercell of the heterostructures. Additionally, graphene-like GaN and ZnO monolayers are pronounced semiconductors. They have been successfully used to form graphene-based vertical heterostructures, which have demonstrated promising electronic and optoelectronic properties [29,30]. The objective of this paper is to consider various ways for controlling the electronic structure of borophene/GaN and borophene/ZnO heterostructures in order to determine the most effective for controlling the type of conductivity of these heterostructures.

## 2. Calculation Details

Calculations of buckled triangular borophene/GaN and buckled triangular borophene/ZnO van der Waals heterostructures were carried out using the DFT method implemented in the Siesta 4.1.5 software package [31,32]. The exchange-correlation effects were described within the generalized gradient approximation (GGA) in the Perdew, Burke, and Ernzerhof (PBE) parameterization [33]. The van der Waals interaction between the layers in the heterostructure was considered using the correction scheme proposed by Grimme [34]. When optimizing the geometry of the structure, we used the split valence DZP basis set. The Brillouin zone sampling was carried out according to the Monkhorst–Pack method [35] with a k-points grid of 10 × 5 × 1. The relaxation of the structure was carried out until the accuracy in terms of value of the forces acting on each atom did not reach 0.04 eV/Å, and in terms of the total energy, did not reach 10^−5^ eV. To minimize the energy of the electronic subsystem, an efficient Broyden–Pulay mixing scheme was used [36]. In order to avoid an interaction between the neighboring structures in the calculation scheme with periodic boundary conditions, the translation vector along the *z*-axis of the supercell was set to be greater than 20 Å. The real-space grid cutoff was chosen to be 300 Ry.

## 3. Results

### 3.1. Atomic and Electronic Structure of Undeformed Borophene/GaN and Borophene/ZnO Van der Waals Heterostructures

**Figure 1:**
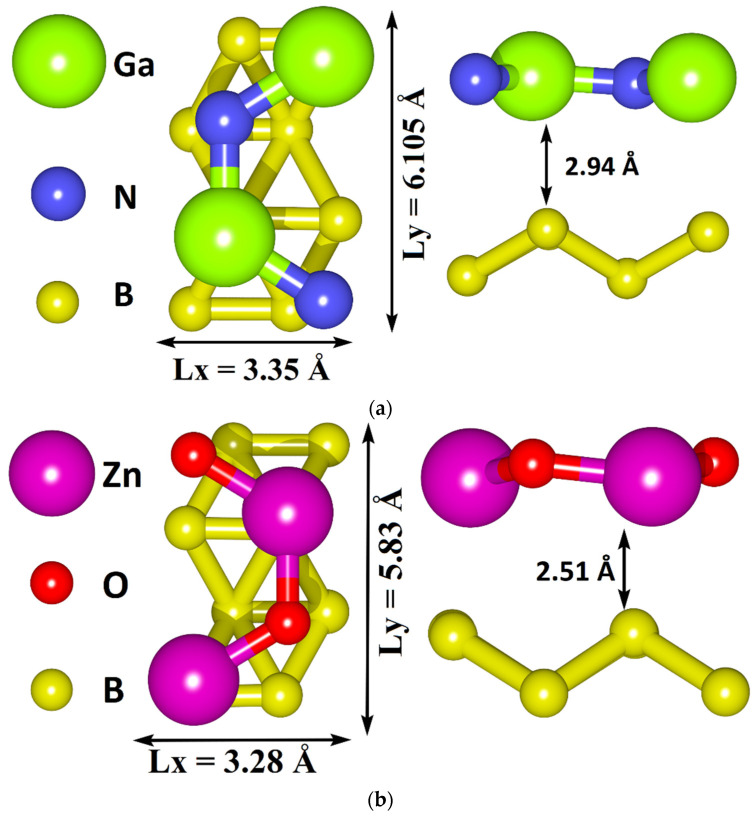
Thermodynamically stable supercells of borophene/GaN (**a**) and borophene/ZnO (**b**) van der Waals heterostructures.

The atomistic models of borophene/GaN and borophene/ZnO heterostructures were built within our previous study and tested for the thermodynamic stability [37]. The thermodynamic stability was estimated from the binding energy E_b_, which was calculated as the difference between the total energy of the heterostructure and the total energy of the isolated monolayers. The binding energy E_b_ was ~−50 meV/atom for the supercell of the borophene/GaN heterostructure and ~−80 meV/atom for the supercell of borophene/ZnO. Figure 1 shows the thermodynamically stable supercells of borophene/GaN and borophene/ZnO van der Waals heterostructures with translation vectors L_x_ and L_y_, as well as the distance between the layers along the Z axis. The supercell translation vectors were L_x_ = 3.35 Å and L = 6.10 Å for the borophene/GaN heterostructure and L_x_ = 3.28 Å and L_y_ = 5.83 Å for the borophene/ZnO heterostructure. The distance between the borophene and GaN monolayers along the Z axis was 2.91 Å, and between the borophene and ZnO monolayers was 2.51 Å.

**Figure 2:**
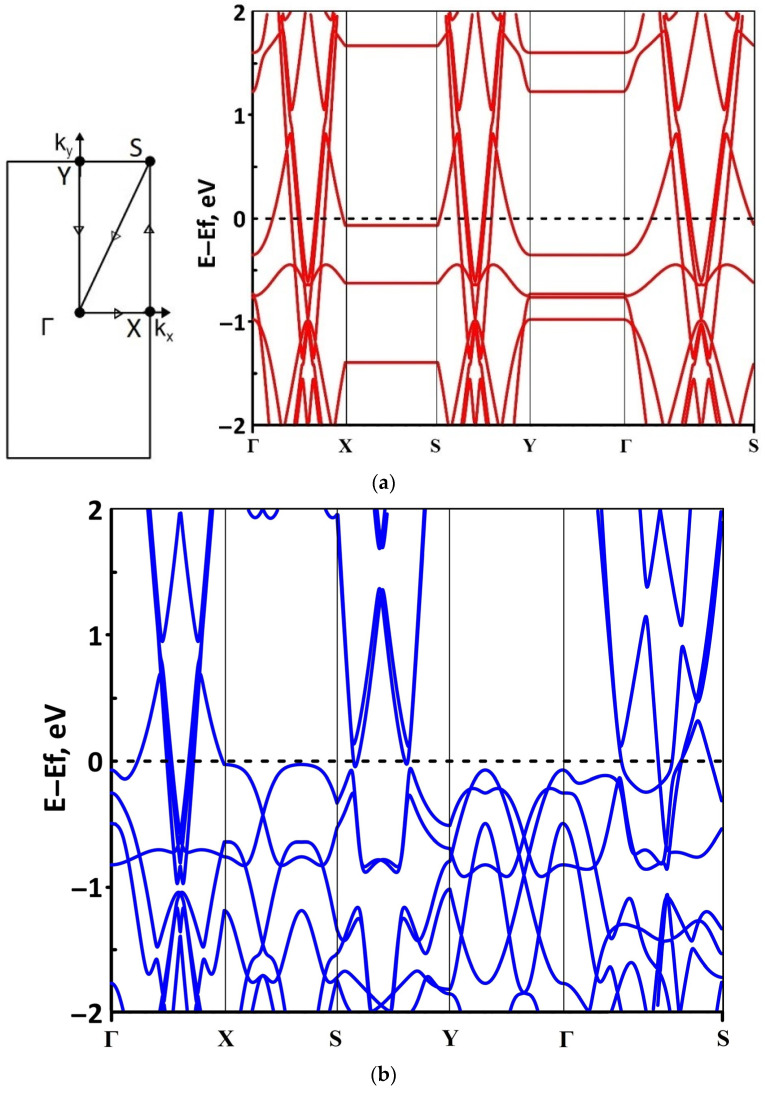
Band diagrams near the Fermi level (shifted to 0 eV) of borophene/GaN (**a**) and borophene/ZnO (**b**) van der Waals heterostructures.

The band structure was calculated for the supercells of the borophene/GaN and borophene/ZnO heterostructures. Figure 2 shows a fragment of the band diagram near the Fermi level, since the electronic states near the Fermi level make a decisive contribution to the electronic and electroconductive properties of the material. The Brillouin zone was a rectangle. The high symmetry path was Γ–X–S–Y–Γ–S. Figure 2a shows that the borophene/GaN heterostructure has a gapless band structure. The edges of the valence and conduction bands touch each other between the Γ–X, S–Y, and Γ–S points of the Brillouin zone (in the direction of the wave vector k_x_). A linear dispersion law is observed between the Γ–X, S–Y, and Γ–S points of the Brillouin zone. Near the valence band maximum (VBM) between the Γ–X, S–Y, and Γ–S points of the Brillouin zone, the dispersion law is close to the isotropic parabolic. Between the X–S and Y–Γ points of the Brillouin zone (in the direction of the wave vector k_y_), the flat energy bands are observed near the VBM and the conduction band minimum (CBM). As can be seen from Figure 2b, there is no energy gap in the band structure of the borophene/ZnO heterostructure. The edges of the valence and conduction bands touch each other between the Γ–X and Γ–S points of the Brillouin zone. A linear dispersion law is observed between the Γ–X points of the Brillouin zone both near the VBM and CBM. In the valence band, the linear dispersion law is replaced by a parabolic one.

**Figure 3:**
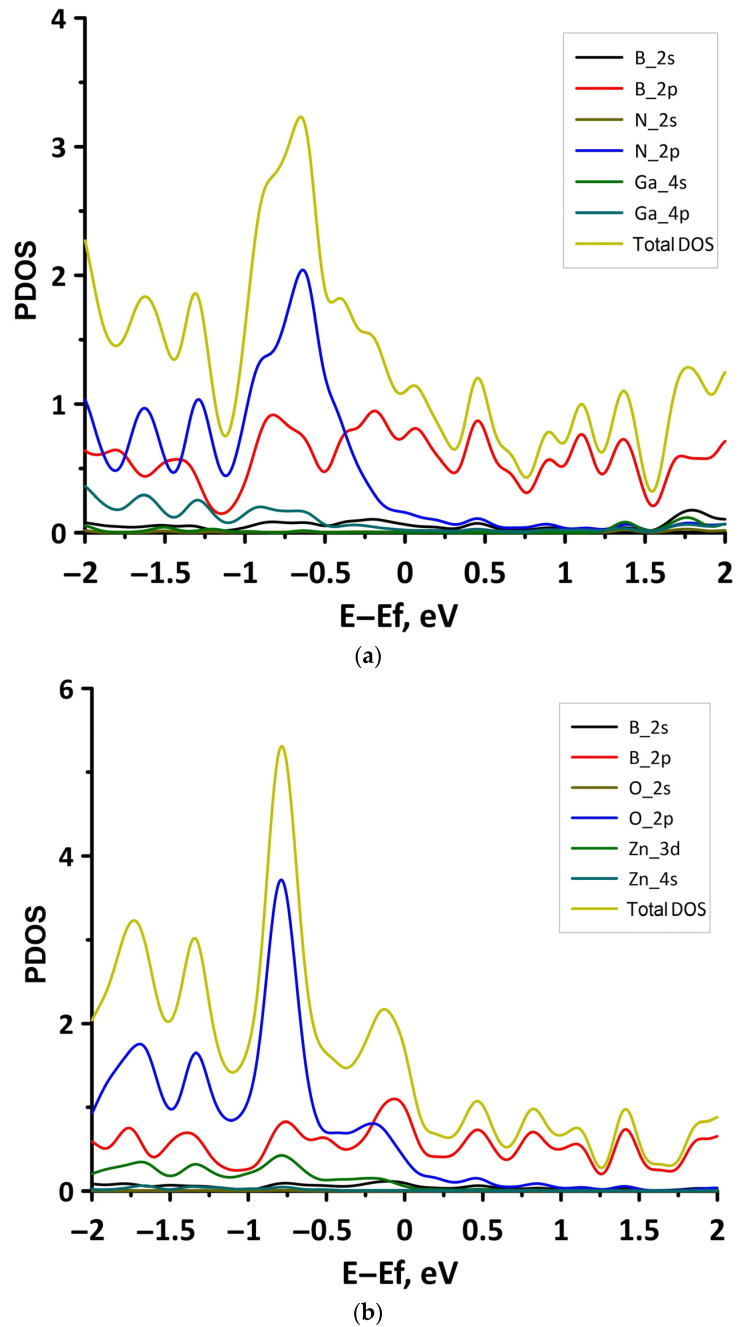
PDOS of borophene/GaN (**a**) and borophene/ZnO (**b**) van der Waals heterostructures.

In order to explain the features of the electronic structure of borophene/GaN and borophene/ZnO van der Waals heterostructures, we analyzed the projected densities of the electronic states (PDOS) distributions. The calculated PDOS distributions are shown in Figure 3. For the borophene/GaN heterostructure (Figure 3a), the electronic states in the valence band (to the left of 0 eV) are mainly formed by the unoccupied 2p-orbitals of the N atoms, while the electronic states in the conduction band (to the right of 0 eV) are formed by the unoccupied 2p-orbitals of the B atoms. For the borophene/ZnO heterostructure (Figure 3b), the electronic states of the valence band are formed by the unoccupied 2p-orbitals of N atoms, and the electronic states of the conduction band are formed by the unoccupied 2p-orbitals of the B atoms. Thus, it is borophene that makes the decisive contribution to the formation of the conduction band of the borophene/GaN and borophene/ZnO heterostructures, explaining their gapless character.

**Figure 4:**
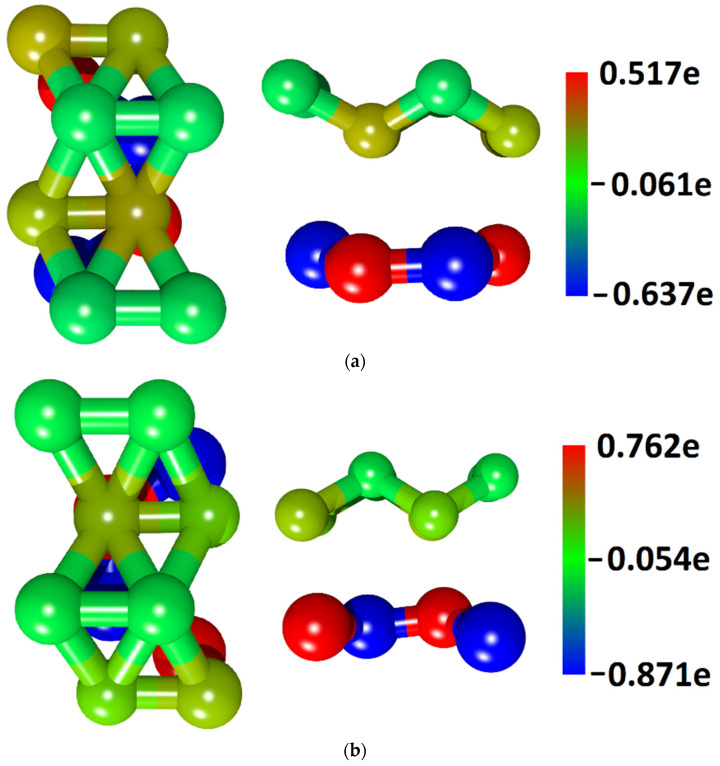
Distribution of electron charge density over atoms of supercells of borophene/GaN (**a**) and borophene/ZnO (**b**) van der Waals heterostructures.

The significant contribution of borophene to the electronic properties of the borophene/GaN and borophene/ZnO heterostructures also follows from the Mulliken population analysis. The calculated electron charge density distributions over the supercell atoms are shown in Figure 4. It can be seen that there is an electronic charge transfer between the GaN and borophene monolayers, as well as between the ZnO and borophene monolayers. The charge is transferred from borophene to the GaN and ZnO monolayers. The total charge transferred from borophene to the GaN monolayer is 0.235 |*e*|, and from borophene to the ZnO monolayer is 0.22 |*e*|. The close values of the transferred charge for both heterostructures are explained by the proximity of Ga and Zn in the fourth row of the periodic table, and N and O in the second row of the periodic table.

**Figure 5:**
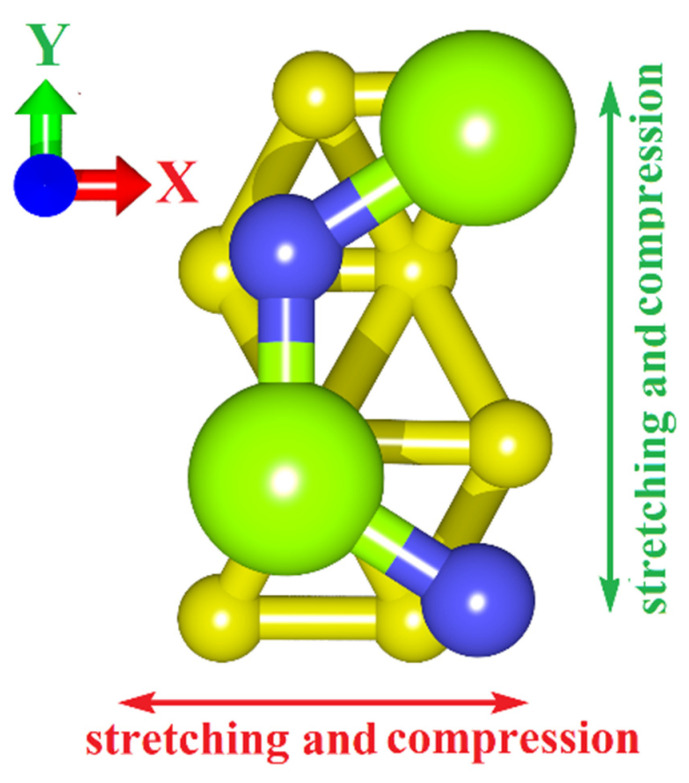
Scheme of stretching/compression of the van der Waals heterostructures under study on the example of a supercell of borophene/ZnO heterostructure.

After analyzing the regularities of the electronic structure of borophene/GaN and borophene/ZnO van der Waals heterostructures, we proceeded to identify the ways to control their electronic properties, including studying the possibility of opening an energy gap in the band structure. As such ways, various types of mechanical deformations were considered, namely, uniaxial and biaxial stretching/compression, which are often used in a real experiment. Figure 5 shows the stretching/compression schemes used in numerical experiments by the example of a supercell of a borophene/GaN heterostructure. The cases of the deformation of the uniaxial compression and stretching (along the Y axis) by 1–14%, deformation of the biaxial compression and stretching (along the X and Y axes) by 1–6% were considered.

The selected ranges of the uniaxial and biaxial deformations are due to the computational complexity of the quantum calculations performed for the heterostructures under study. At the same time, the problem of determining the ultimate strength of borophene/GaN and borophene/ZnO van der Waals heterostructures under stretching/compression was not solved in this paper.

### 3.2. Effect of Stretching/Compression on the Electronic Structure of Borophene/GaN and Borophene/ZnO Van der Waals Heterostructures

#### 3.2.1. Uniaxial Deformation

**Figure 6:**
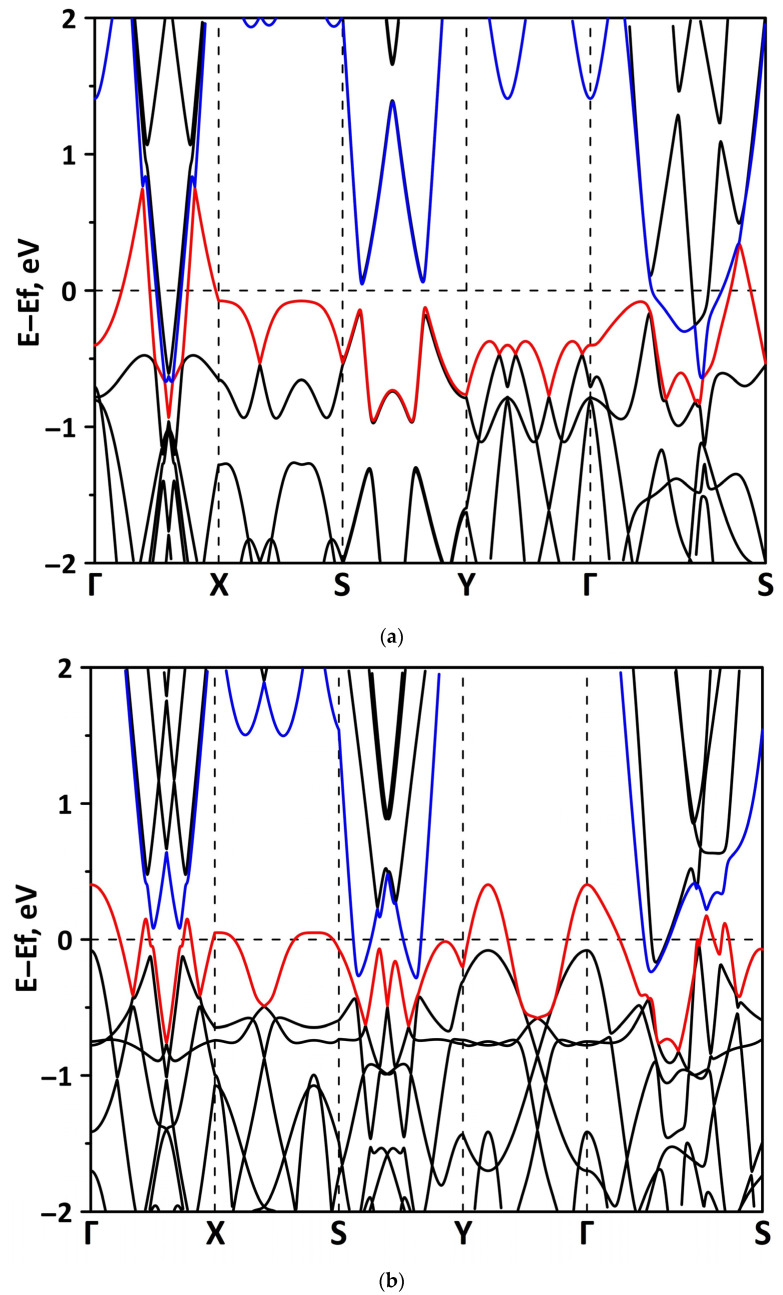
Fragments of the band diagrams near the Fermi level (shifted to 0 eV) of borophene/GaN heterostructure under uniaxial compression by 1% (**a**) and 14% (**b**). The VBM is highlighted in red and the CBM is highlighted in blue.

**Figure 7:**
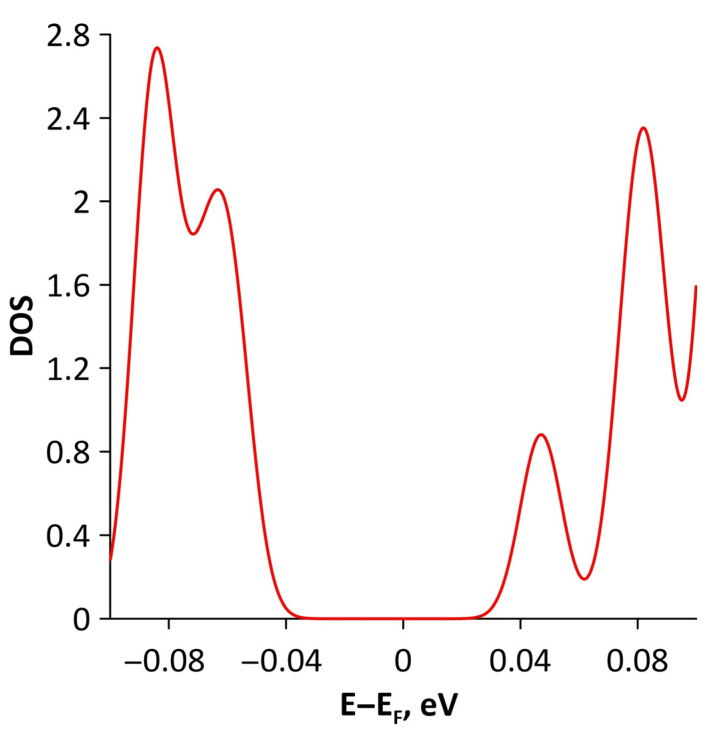
Fragment of DOS near the Fermi level (shifted to 0 eV) of borophene/GaN heterostructure under uniaxial compression by 14%.

Changes in the electronic structure of borophene/GaN and borophene/ZnO van der Waals heterostructures under stretching/compression were analyzed based on the calculated band structure and DOS. Let us turn to the analysis of the calculated data for the borophene/GaN heterostructure. The band diagrams of the borophene/GaN heterostructure under uniaxial compression are shown in Figure 6. Here, and below, we will present the band diagrams for two cases: small (1%) and maximum (14%) deformation within the framework of our numerical experiments. This was done in order to clearly demonstrate the degree of influence of deformation on the electronic structure of the heterostructures under study. Comparing with the band diagram of an undeformed borophene/GaN heterostructure (Figure 2a), it can be noted that even at small deformations, the pattern of the energy subbands begins to change rather strongly. The energy bands near the VBM and CBM between the X–S and Y–Γ points of the Brillouin zone cease to be flat and exhibit a dispersion law close to parabolic (Figure 6a). This fact indicates a decrease in the carrier mobility in the direction of the wave vector k_y_ as compared to an undeformed structure. Consequently, under uniaxial compression, the contribution of the GaN monolayer to electronic structure of the borophene/GaN heterostructure becomes more significant. This is especially noticeable for the case of compression by 14% (Figure 6b). It can be seen that between the Γ–X, S–Y, and Γ–S points of the Brillouin zone, an energy gap appears between the VBM (highlighted in red) and CBM (highlighted in blue). In order to find out whether the energy gap opens in the band structure, we calculated the DOS distribution for this case. A fragment of this distribution near the Fermi level is shown in Figure 7. It can be seen that an interval with a zero-electron density of 0.028 eV has appeared near the Fermi level.

**Figure 8:**
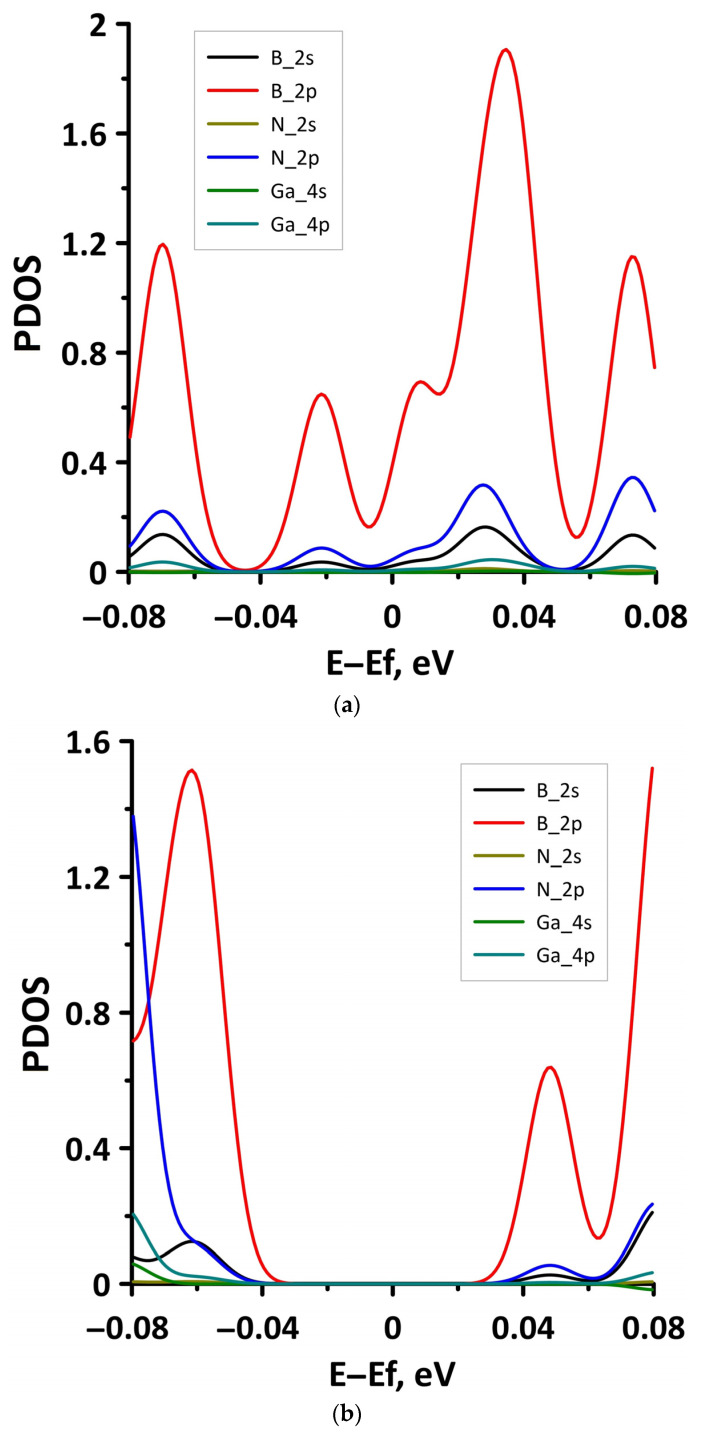
Fragments of PDOS near the Fermi level (shifted to 0 eV) of borophene/GaN heterostructure under uniaxial compression by 1% (**a**) and 14% (**b**).

To explain the reason for the opening of the energy gap in the band structure of the borophene/GaN heterostructure, we present the graphs of the projected DOS (PDOS) for the cases of compression by 1% and 14% (Figure 8). It can be seen that in the case of compression by 1% (Figure 8a), the PDOS of the 2s- and 2p-orbitals of the B atoms, as well as the 2p-orbitals of the N atoms, contain electronic states near the Fermi level (at 0 eV). In the case of compression by 14%, there are no electronic states in the PDOS of 2s- and 2p-orbitals of the B atoms, 2s- and 2p-orbitals of the N atoms, and 4s- and 4p-orbitals of the Ga atoms near the Fermi level. This is precisely the reason for the opening of the energy gap in the band structure of the borophene/GaN heterostructure.

**Figure 9:**
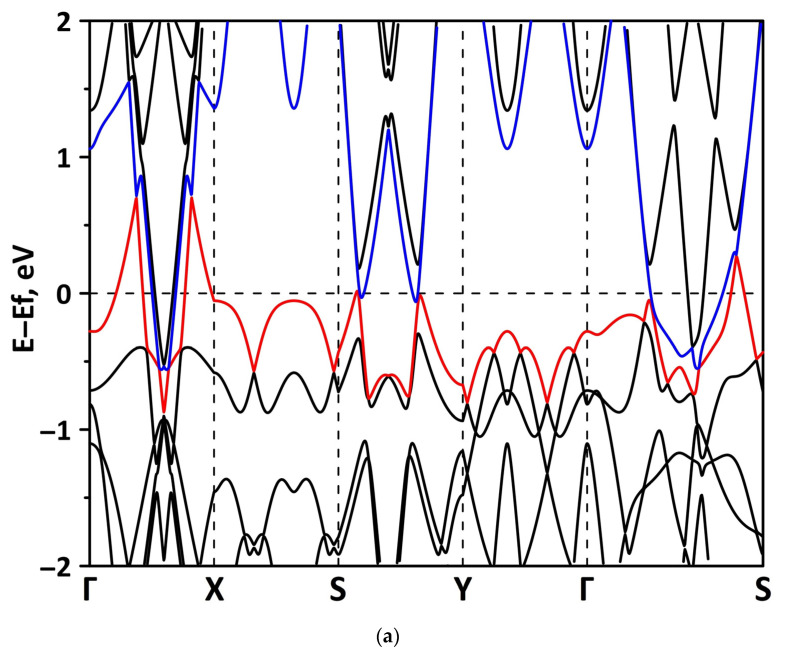

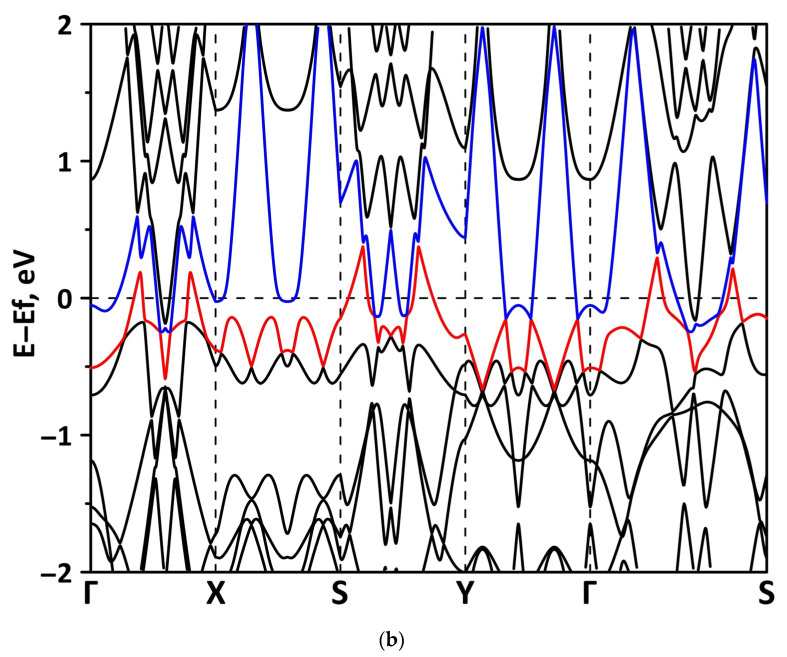
Fragments of the band diagrams near the Fermi level (shifted to 0 eV) of borophene/GaN heterostructure under uniaxial stretching by 1% (**a**) and 14% (**b**). The VBM is highlighted in red and the CBM is highlighted in blue.

**Figure 10:**
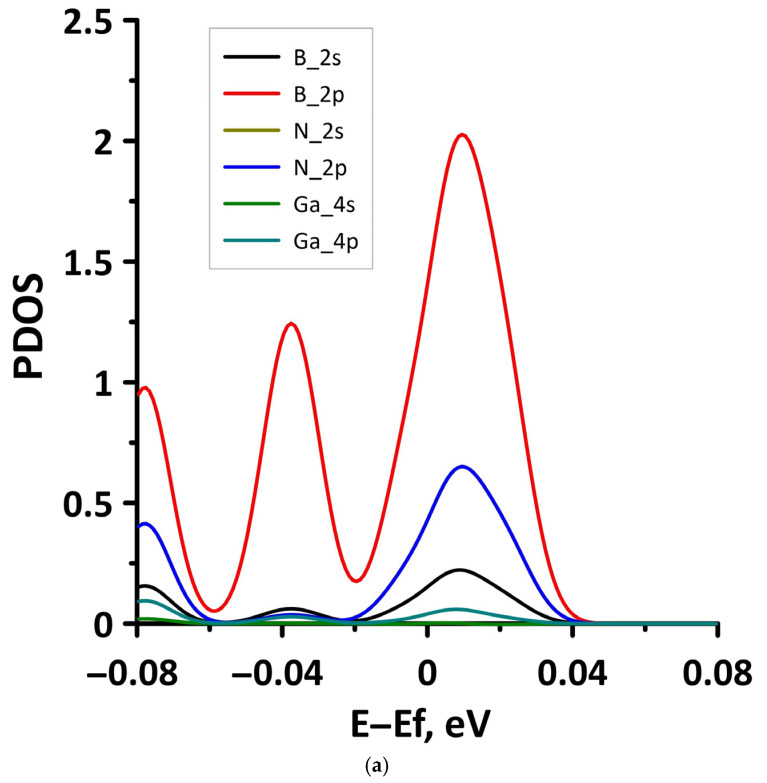

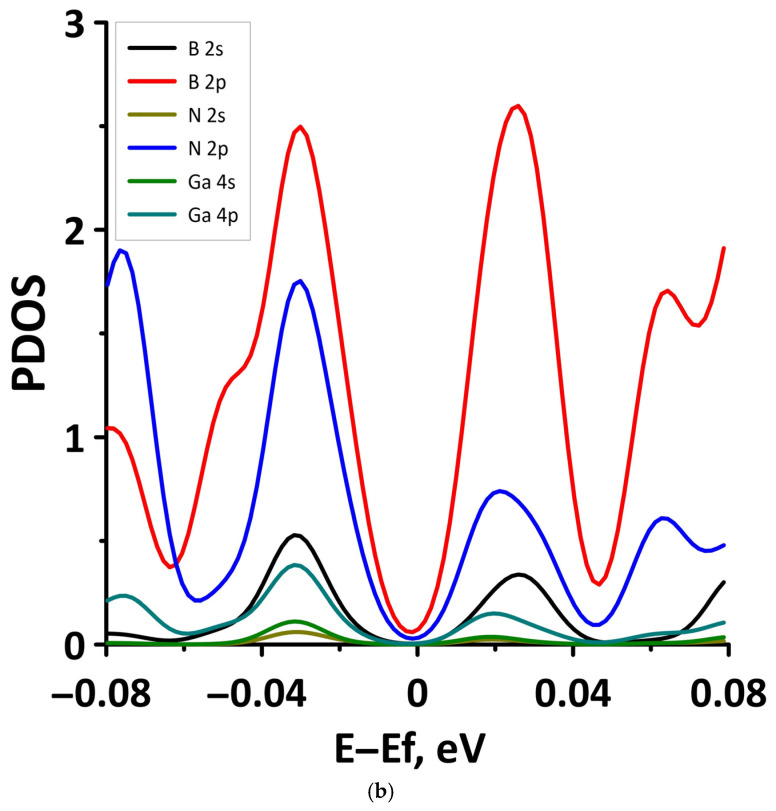
Fragments of PDOS near the Fermi level (shifted to 0 eV) of borophene/GaN heterostructure under uniaxial stretching by 1% (**a**) and 14% (**b**).

The band diagrams of the borophene/GaN heterostructure at minimum (1%) and maximum (14%) uniaxial stretching are shown in Figure 9. It can be seen that the flat energy subbands in the direction of the wave vector k_y_ (between the X–S and Y–Γ points of the Brillouin zone) change to parabolic ones, as in the case of a uniaxial compression (Figure 6). In the direction of the wave vector k_x_, as the stretch increases, the energy subbands split with the formation of a larger number of peaks. In this case, the edges of the valence and conduction bands not only touch in the direction of the wave vector k_x_, but also in the direction of the wave vector k_y_ (between the Y–Γ points of the Brillouin zone). The calculated PDOS distributions clearly explain the gapless band structure of the borophene/GaN heterostructure under uniaxial stretching. The fragments of these distributions near the Fermi level are shown in Figure 10. It can be seen that a nonzero DOS near the Fermi level is typical for the 2p-orbitals of the B atoms and 2p-orbitals of N atoms both at the minimum (1%) and maximum stretching (14%). At the same time, it cannot be said that an increase in stretching has no effect on the PDOS near the Fermi level. In the case of uniaxial stretching by 14%, noticeable peaks at the Fermi level in the PDOS of the 2p-orbitals of the B atoms and the 2p-orbitals of the N atoms are replaced by troughs with DOS values close to zero. In addition, the PDOS of the 2s-orbitals of the B atoms vanishes at the Fermi level. However, to open the energy gap, the deforming force in the considered range of values turned out to be insufficient.

**Figure 11:**
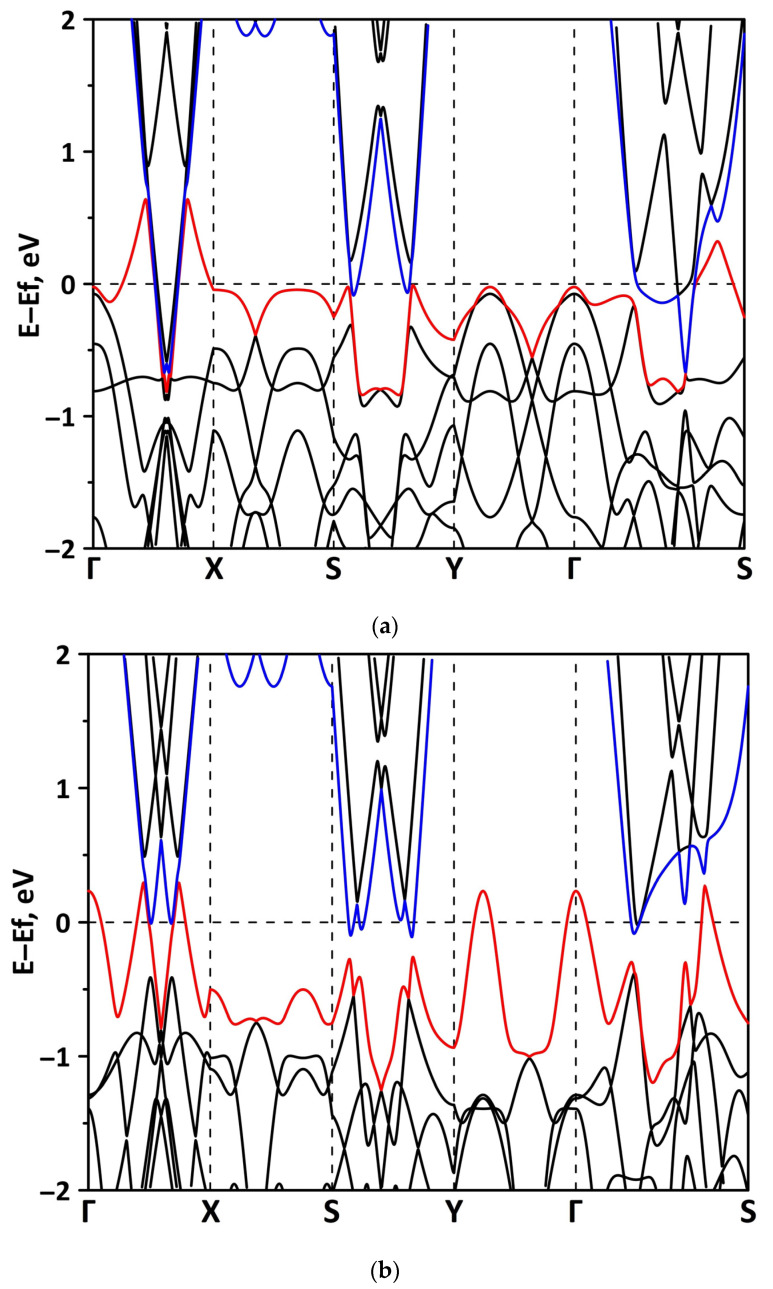
Fragments of the band diagrams near the Fermi level (shifted to 0 eV) of borophene/ZnO heterostructure under uniaxial compression by 1% (**a**) and 14% (**b**). The VBM is highlighted in red and the CBM is highlighted in blue.

Let us turn to the consideration of the band diagrams of the borophene/ZnO heterostructure under uniaxial compression and stretching. The cases of minimum (1%) and maximum (14%) uniaxial compression are shown in Figure 11. It can be noted that the band diagram of the borophene/ZnO heterostructure at a minimal compression (1%) almost does not change in comparison with the band diagram of the undeformed heterostructure. The only difference is that, at compression by 1%, the edges of the valence and conduction bands almost touch each other between the S–Y points of the Brillouin zone. At maximum compression, changes in the band diagram become more noticeable. Between the S–Y and Γ–S points of the Brillouin zone, a rather noticeable energy gap appears between the edges of the valence and the conduction bands. Between the Γ–X points of the Brillouin zone, the edges of the valence and conduction bands still touch each other, but without the formation of Dirac cones, which are characteristic of borophene.

**Figure 12:**
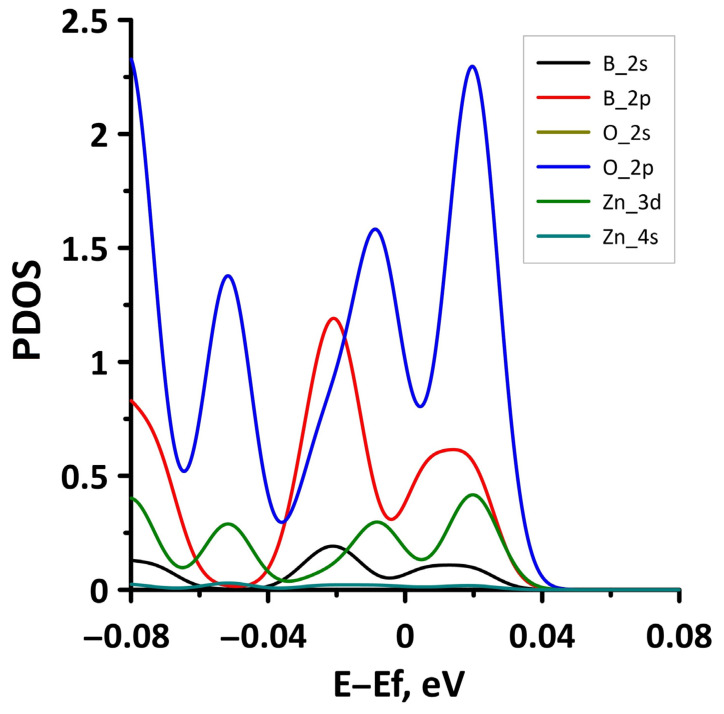
Fragments of PDOS near the Fermi level (shifted to 0 eV) of borophene/ZnO heterostructure under uniaxial compression by 14%.

We present the PDOS distribution (Figure 12) in the case of maximum compression (14%) in order to explain the absence of an energy gap in the band structure of the borophene/ZnO heterostructure. The plot in Figure 12 clearly shows that zero DOS values near the Fermi level are present only in the PDOS distributions of the 4s-orbitals of the Zn atoms. The PDOS distributions for both the occupied and unoccupied orbitals of the B and O atoms have a nonzero density of the states near the Fermi level. Thus, the presence of a nonzero DOS near the Fermi level in the PDOS of B atoms in borophene and N atoms in ZnO results in a gapless band structure of the borophene/ZnO heterostructure.

**Figure 13:**
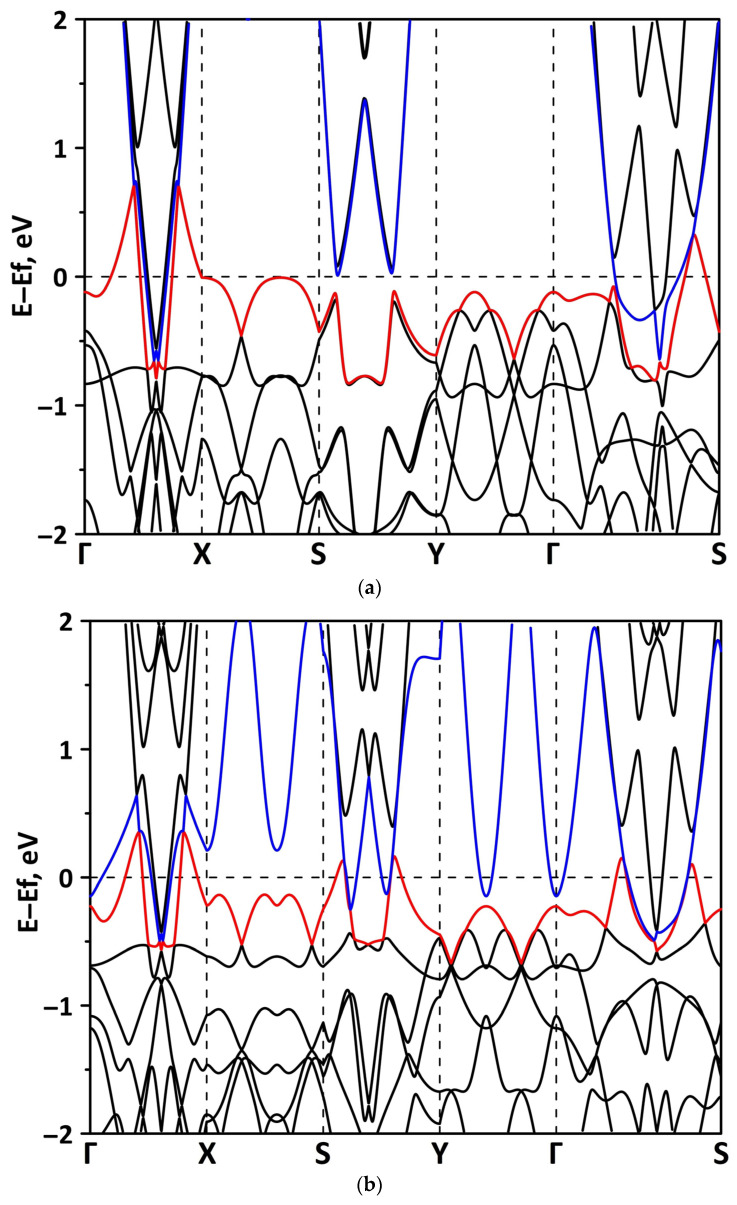
Fragments of the band diagrams near the Fermi level (shifted to 0 eV) of borophene/ZnO heterostructure under uniaxial stretching by 1% (**a**) and 14% (**b**). The VBM is highlighted in red and the CBM is highlighted in blue.

**Figure 14:**
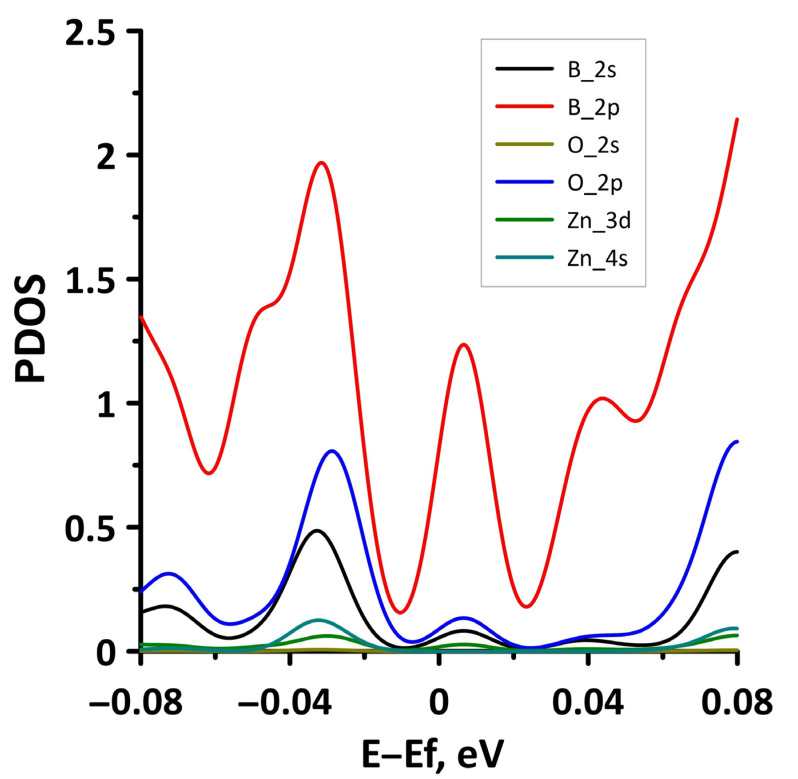
Fragments of PDOS near the Fermi level (shifted to 0 eV) of borophene/ZnO heterostructure under uniaxial stretching by 14%.

The band diagrams of the borophene/ZnO heterostructures at minimal (1%) and maximum (14%) uniaxial stretching are shown in Figure 13. It can be seen that the band diagram of the borophene/ZnO heterostructure at minimal stretching remains unchanged as compared to the undeformed heterostructure. At maximum stretching, noticeable changes are observed at the CBM. In particular, energy subbands appear near the Fermi level in the direction of the wave vector k_y_ between the X–S and Y–Γ points of the Brillouin zone. In the direction of the wave vector k_x_, the edges of the valence and conduction bands touch not only between the Γ–X points (as in the case of stretching by 1%), but also between the S–Y points of the Brillouin zone. Both of these changes in the conduction band of the borophene/ZnO heterostructure upon stretching by 14% can be explained from the PDOS plot shown in Figure 14. It can be seen that there is a peak in the DOS distribution of the unoccupied 2p-orbitals of the B atoms near the Fermi level. According to Figure 3b, it is the 2p-orbitals of the B atoms that make the decisive contribution to the formation of the conduction band of the borophene/ZnO heterostructure.

**Figure 15:**
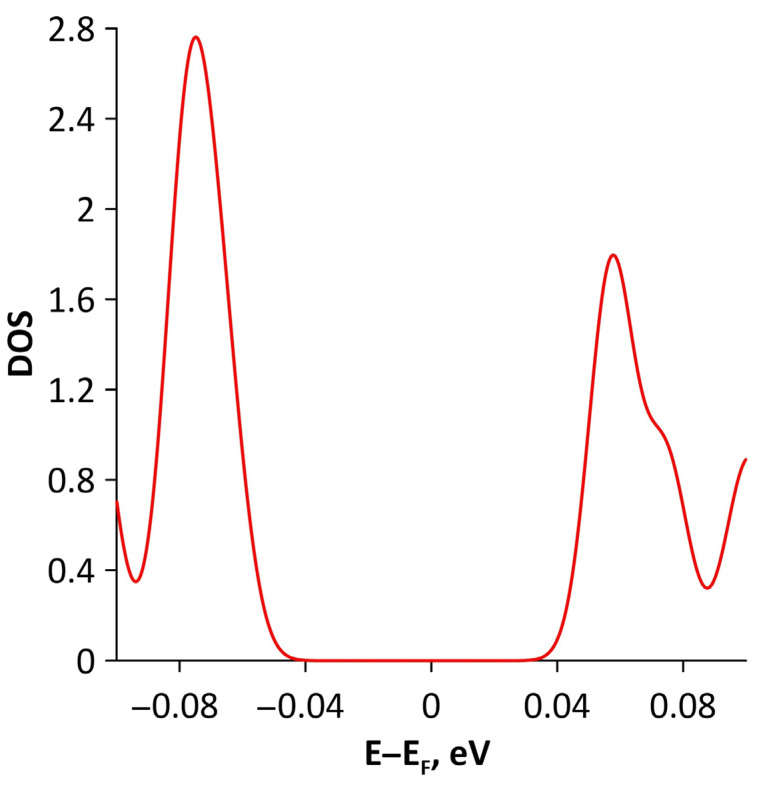
Fragment of DOS near the Fermi level (shifted to 0 eV) of borophene/ZnO heterostructure under uniaxial stretching by 10%.

**Figure 16:**
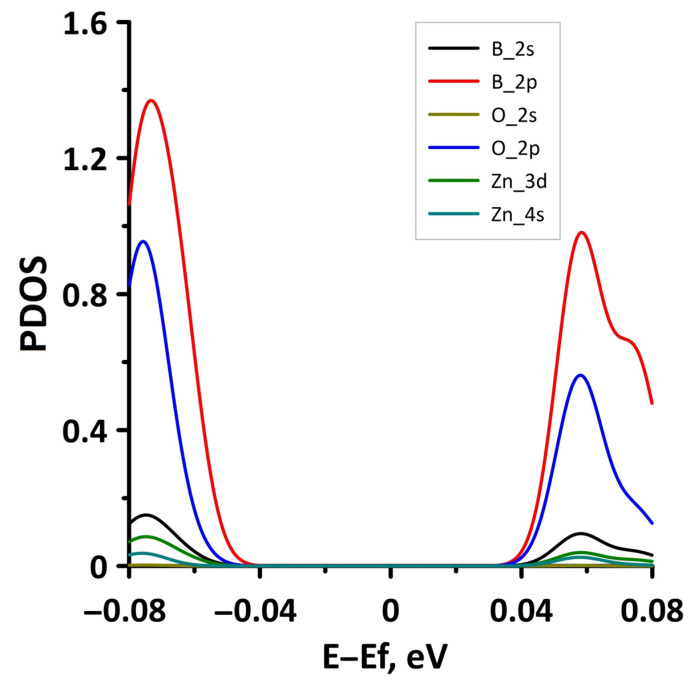
Fragments of PDOS near the Fermi level (shifted to 0 eV) of borophene/ZnO heterostructure under uniaxial stretching by 10%.

The opening of the energy gap between the valence and conduction bands in the band structure of the borophene/ZnO heterostructure was found for the case of stretching by 10%. Since the energy gap is quite small, we present a fragment of the DOS distribution near the Fermi level to show it more clearly (Figure 15). The plot in Figure 15 demonstrates that the size of the energy gap is 0.063 eV. An explanation of the mechanism for the appearance of an energy gap in the band structure of the borophene/ZnO heterostructure can be given by the PDOS near the Fermi level. This plot is shown in Figure 16. It can be seen that the PDOS of the s- and p-orbitals of the borophene atoms and ZnO atoms have a zero density of states near the Fermi level. Consequently, each orbital contributes to the opening of the energy gap between the valence and conduction bands.

Thus, we can conclude that the borophene/GaN and borophene/ZnO heterostructures react differently to different types of uniaxial deformation. The borophene/GaN heterostructure is more sensitive to uniaxial compression, while the borophene/ZnO heterostructure is more sensitive to uniaxial stretching.

#### 3.2.2. Biaxial Deformation

**Figure 17:**
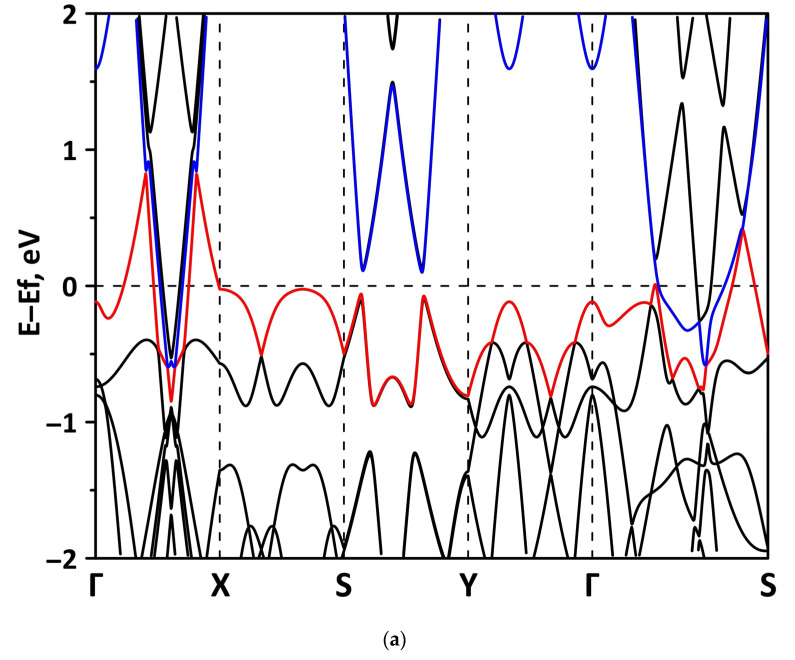

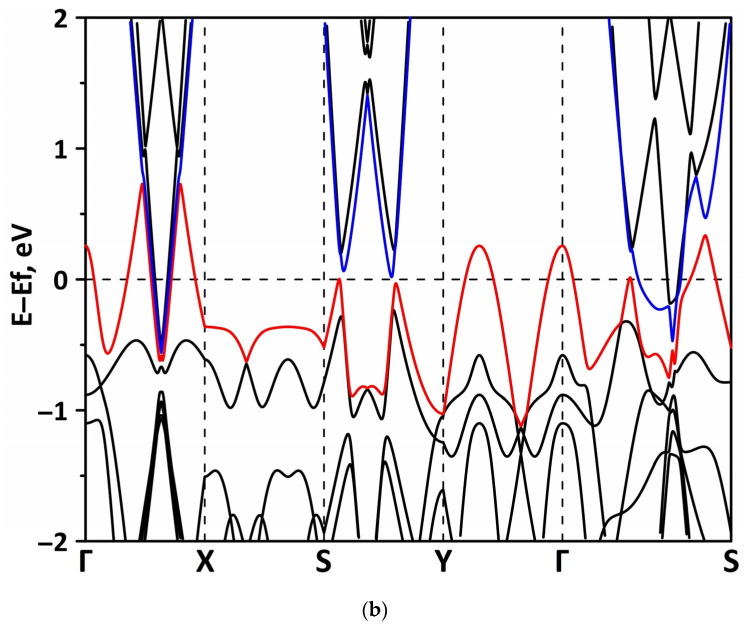
Fragments of the band diagrams near the Fermi level (shifted to 0 eV) of borophene/GaN heterostructure under biaxial compression by 2% (**a**) and 6% (**b**). The VBM is highlighted in red and the CBM is highlighted in blue.

**Figure 18:**
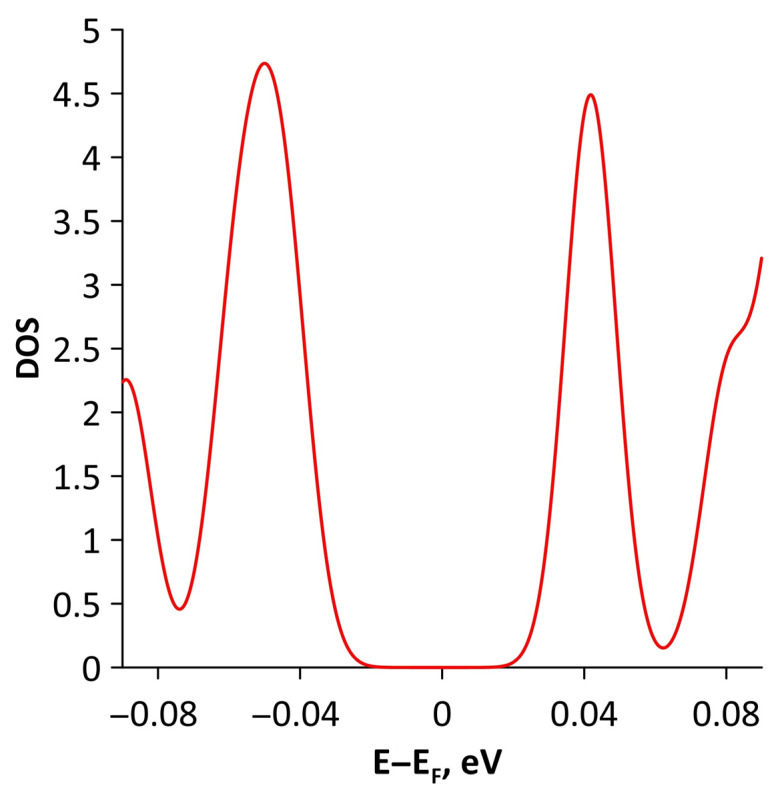
Fragment of DOS near the Fermi level (shifted to 0 eV) of borophene/GaN heterostructure under biaxial compression by 4%.

Next, we turned to the consideration of the biaxial deformation of the borophene/GaN and borophene/ZnO heterostructures. Figure 17 shows the band diagrams of the borophene/GaN heterostructure for the cases of biaxial compression by 2% and 6%. Here, and below, these cases were chosen in order to show whether an increase in the deformation by several times leads to a significant difference in the nature of the change in the band structure of the heterostructure under study. It can be seen that an increase in the degree of biaxial compression by a factor of 3 causes the following changes in the band diagram of the borophene/GaN heterostructure: (1) the Fermi level crosses several times the top of the valence band between the Y–Γ points of the Brillouin zone; and (2) between the Γ–S points of the Brillouin zone, the edges of the valence and conduction bands do not touch each other. It was found that the energy gap between the valence and conduction bands appears at a biaxial compression of 4%. Its value is 0.018 eV. A visual representation of the presence of an energy gap and its size is given by a fragment of the DOS distribution shown in Figure 18.

**Figure 19:**
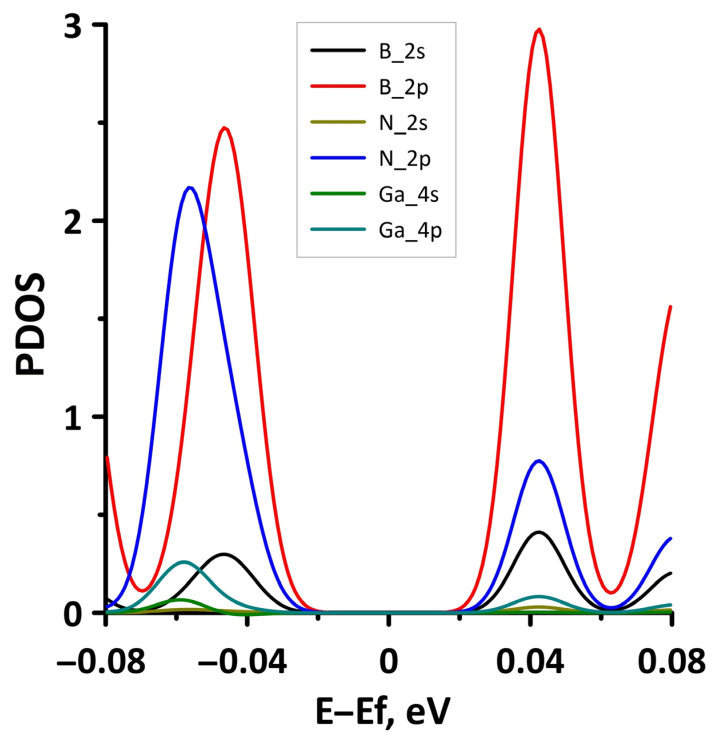
Fragments of PDOS near the Fermi level (shifted to 0 eV) of borophene/GaN heterostructure under biaxial compression by 4%.

**Figure 20:**
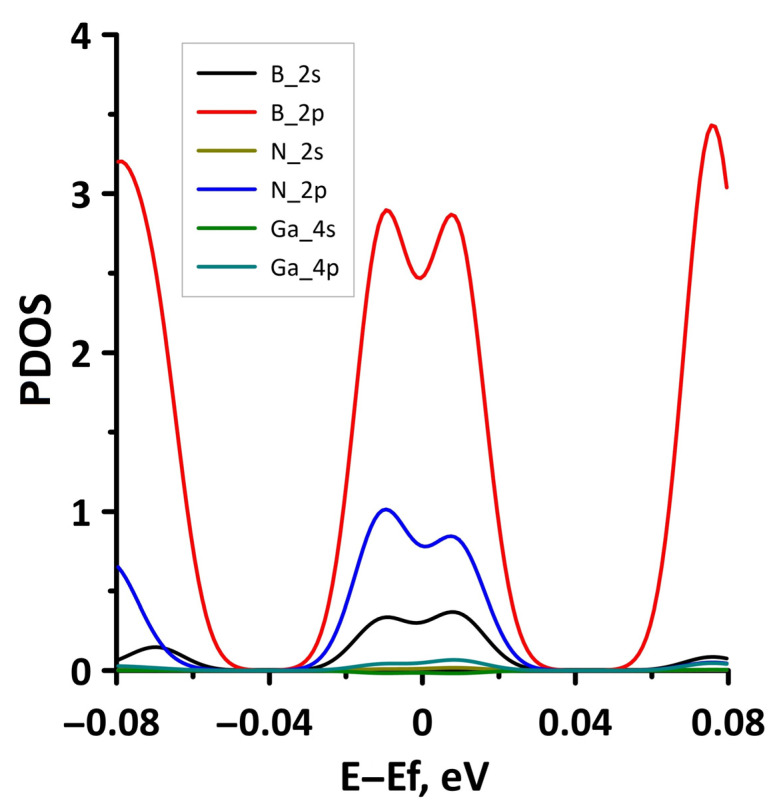
Fragments of PDOS near the Fermi level (shifted to 0 eV) of borophene/GaN heterostructure under biaxial compression by 2%.

In order to understand the reasons for the appearance of the energy gap in the band structure of the borophene/GaN heterostructure, let us analyze the distributions of the PDOS, as for the uniaxial deformations. Figure 19 shows the PDOS for the case of a 4% biaxial compression when there is an energy gap. For comparison, Figure 20 shows the PDOS for a 2% biaxial compression when there is no energy gap. Comparing the PDOS plots in Figure 19 and Figure 20, it can be noted that for the appearance of an energy gap, it is necessary that the zero DOS be present in the distributions of the PDOS near the Fermi level for the s- and p-orbitals of the B, N, and Ga atoms.

**Figure 21:**
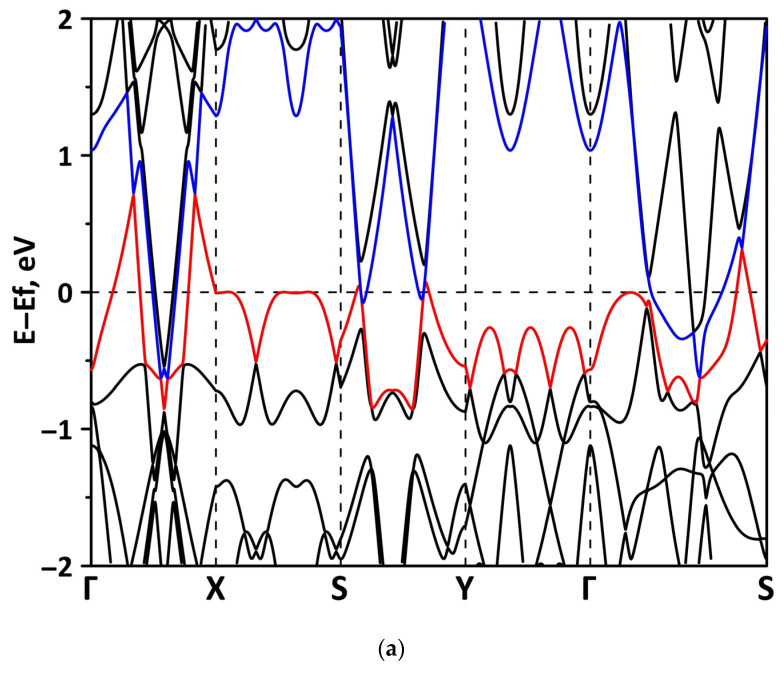

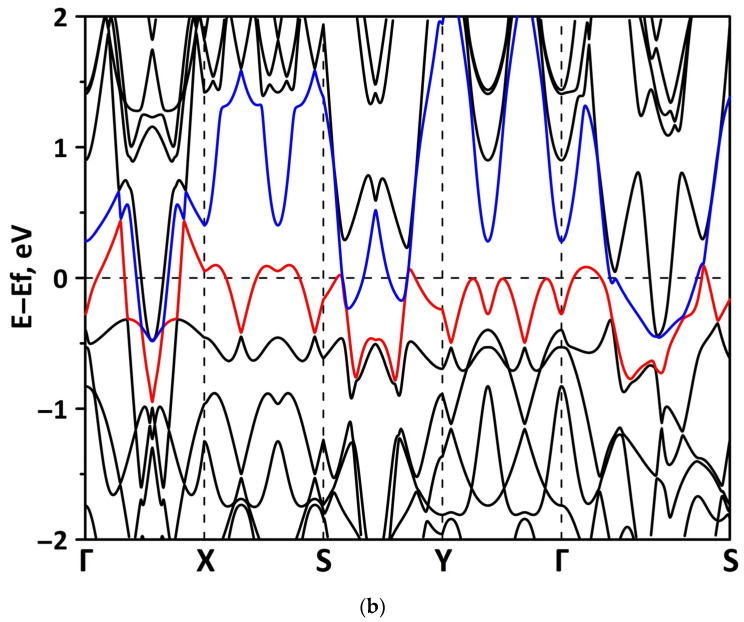
Fragments of the band diagrams near the Fermi level (shifted to 0 eV) of borophene/GaN heterostructure under biaxial stretching by 2% (**a**) and 6% (**b**). The VBM is highlighted in red and the CBM is highlighted in blue.

The band diagrams of the borophene/GaN heterostructure at biaxial stretching by 2% (small deformation) and 6% (maximum deformation in the framework of performed calculations) are shown in Figure 21. It can be seen that a threefold increase in the deformation affected only the density of the energy subbands near the VBM and CBM. In this case, the edges of the valence and conduction bands touch each other between Γ–X, S–Y, and Γ–S points of the Brillouin zone, which indicates the absence of an energy gap in the band structure of the borophene/GaN heterostructure under biaxial stretching.

**Figure 22:**
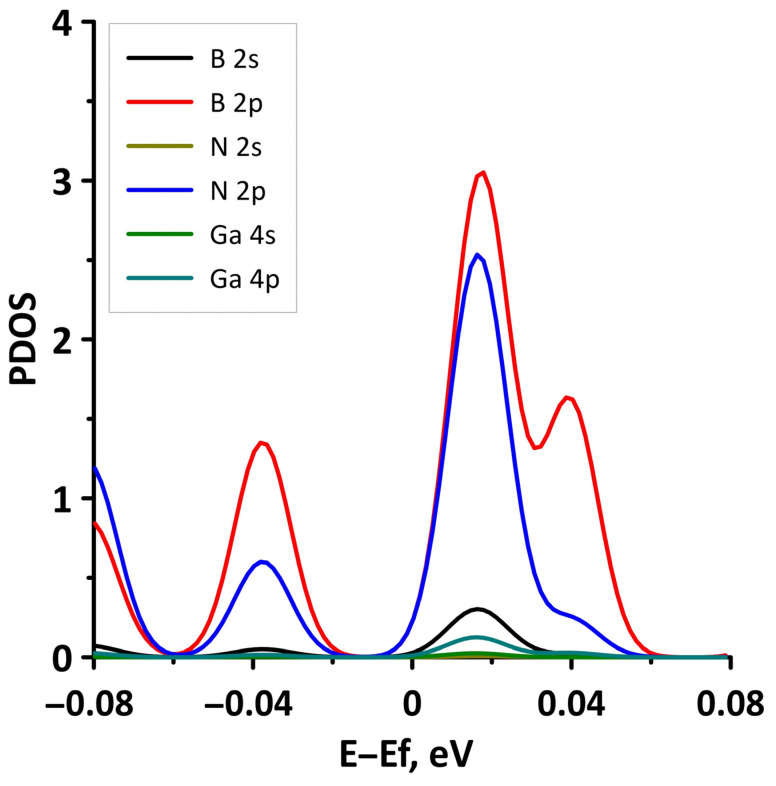
Fragments of PDOS near the Fermi level (shifted to 0 eV) of borophene/GaN heterostructure under biaxial stretching by 6%.

The PDOS plot for the case of 6% biaxial stretching (Figure 22) shows that the PDOS of the p-orbitals of the B atoms and the p-orbitals of the N atoms have a peak near the Fermi level, which also indicates the gapless nature of the band structure of the borophene/GaN heterostructure.

**Figure 23:**
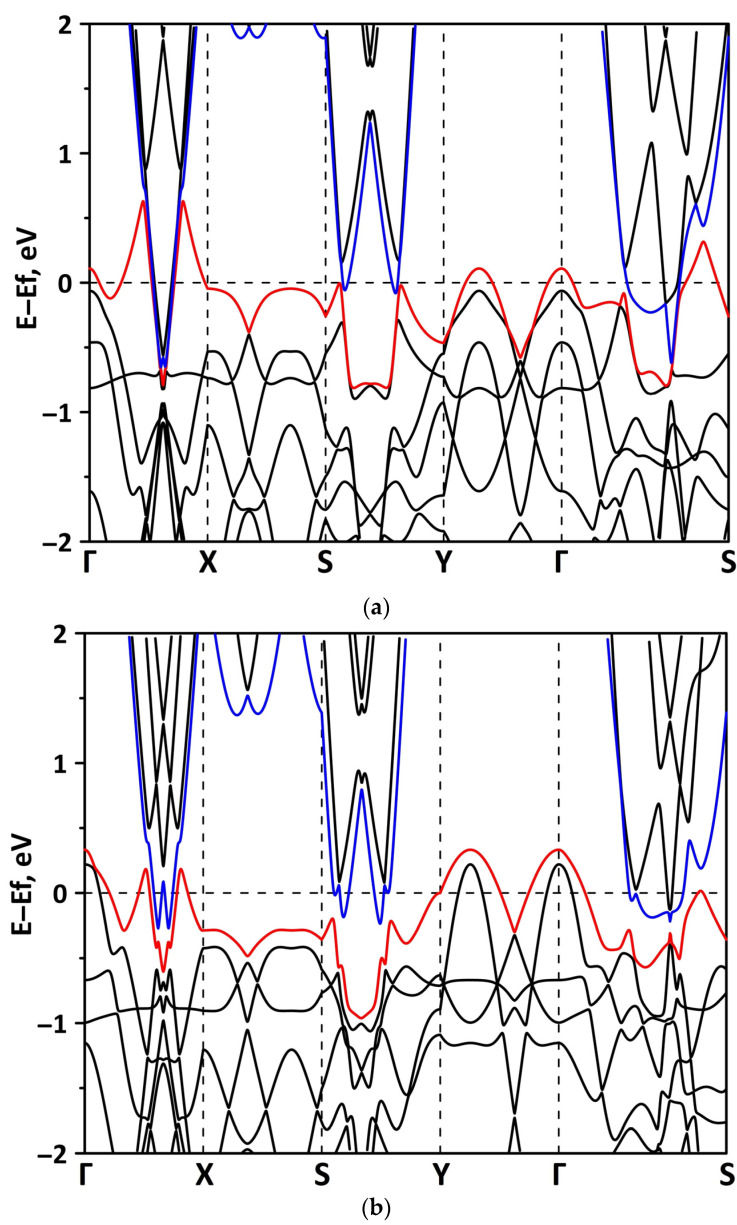
Fragments of the band diagrams near the Fermi level (shifted to 0 eV) of borophene/ZnO heterostructure under biaxial compression by 2% (**a**) and 6% (**b**). The VBM is highlighted in red and the CBM is highlighted in blue.

**Figure 24:**
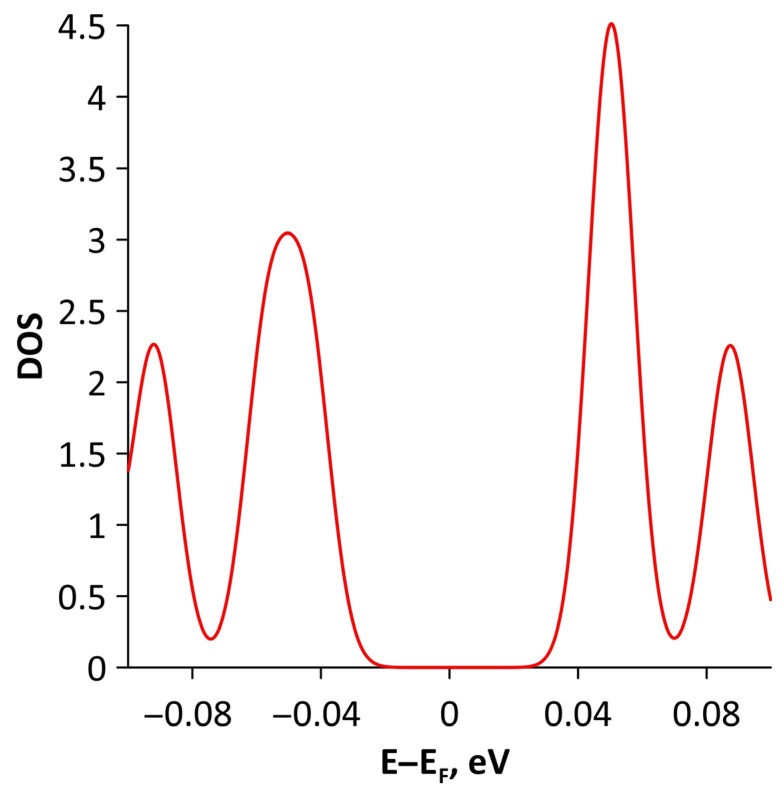
Fragment of DOS near the Fermi level (shifted to 0 eV) of borophene/ZnO heterostructure under biaxial compression by 6%.

**Figure 25:**
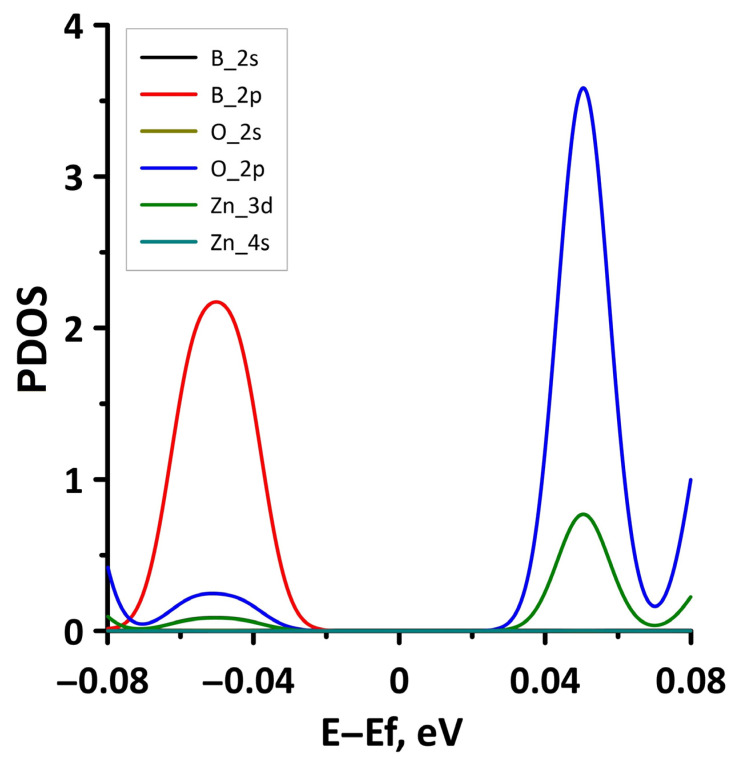
Fragments of PDOS near the Fermi level (shifted to 0 eV) of borophene/ZnO heterostructure under biaxial compression by 6%.

Let us consider the effect of biaxial compression/stretching on the electronic structure of the borophene/ZnO heterostructure. Figure 23 demonstrates the band diagrams for the cases of biaxial compression by 2% and 6%. It can be seen that a threefold increase in the deformation leads to splitting of the energy subbands between the Γ and X points of the Brillouin zone, on the one hand, and the appearance of an energy gap between Γ–X, S–Y, and Γ–S points of the Brillouin zone, on the other hand. The presence of an energy gap in the band structure of the borophene/ZnO heterostructure under biaxial compression by 6% is confirmed by a fragment of the DOS presented in Figure 24. The size of the interval with zero DOS near the Fermi level is 0.012 eV. Figure 25 shows the PDOS of borophene/ZnO heterostructure for this case of biaxial compression. It clearly demonstrates that the appearance of an energy gap in the band structure of the borophene/ZnO heterostructure is achieved due to the presence of zero DOS in the PDOS distributions of the s- and p-orbitals of the B and N atoms, as well as the s- and d-orbitals of the Zn atoms.

**Figure 26:**
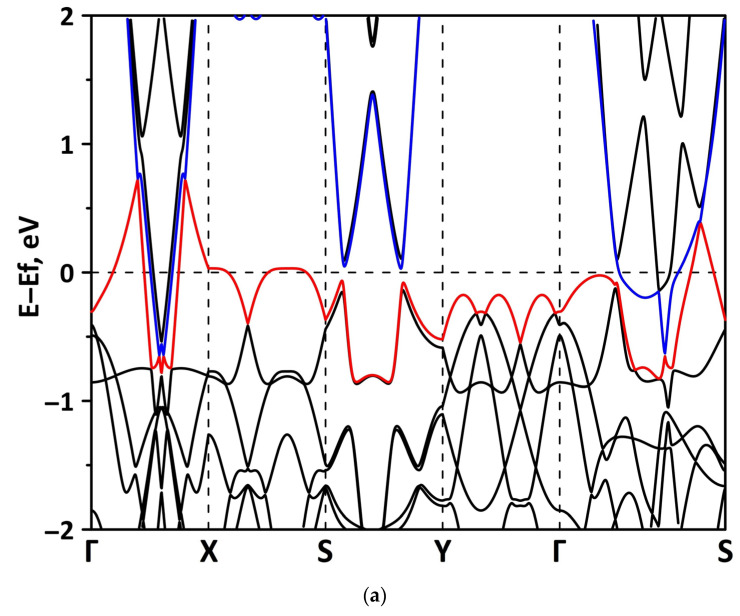

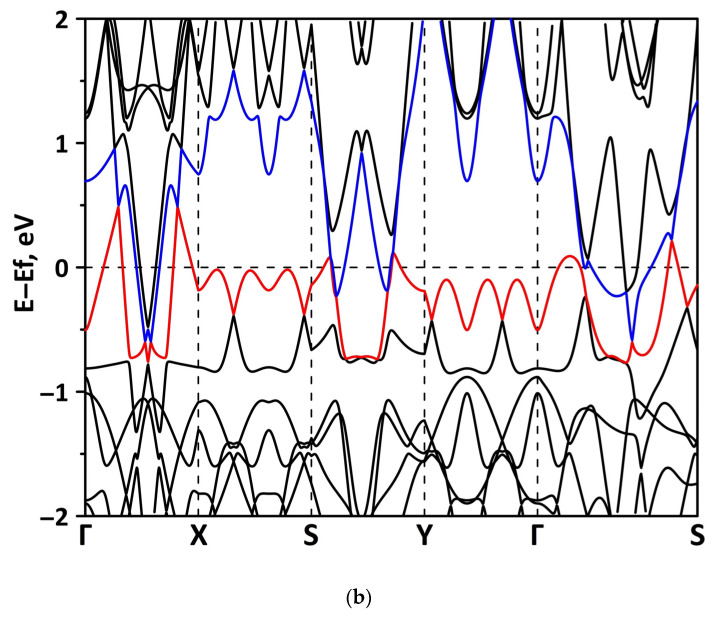
Fragments of the band diagrams near the Fermi level (shifted to 0 eV) of borophene/ZnO heterostructure under biaxial stretching by 2% (**a**) and 6% (**b**). The VBM is highlighted in red and the CBM is highlighted in blue.

**Figure 27:**
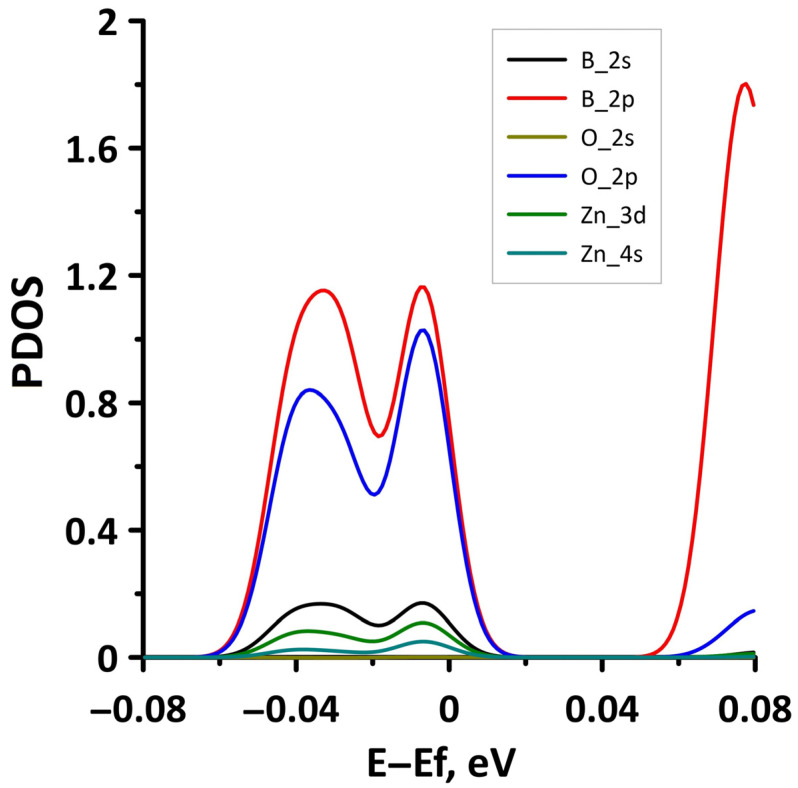
Fragments of PDOS near the Fermi level (shifted to 0 eV) of borophene/ZnO heterostructure under biaxial stretching by 2%.

The band diagrams of the borophene/ZnO heterostructure for cases of 2% and 6% biaxial stretching are shown in Figure 26. It can be seen that a threefold increase in the deformation leads to the appearance of additional energy subbands in the conduction band between the X–S and Y–Γ points of the Brillouin zone and the intersection between the edges of the valence and the conduction bands between the S and Y points of the Brillouin zone. The opening of the energy gap in the band structure of the borophene/ZnO heterostructure did not occur in the considered range of biaxial stretching. Figure 27 shows the PDOS of the borophene/ZnO heterostructure at 2% biaxial stretching. It can be seen that only the s-orbitals of the O atoms have a zero DOS near the Fermi level, while the PDOS of the p-orbitals of the B and O atoms have characteristic peaks of an almost equal intensity near the Fermi level.

Thus, it was found that of the two considered types of biaxial deformation, the two-basic biaxial compression deformation is more effective in terms of controlling the electronic structure of the studied van der Waals heterostructures.

## 4. Discussion

Based on the first-principles calculations, it was found that borophene/GaN and borophene/ZnO heterostructures demonstrate the different sensitivity of the electronic structure to consider the types of axial deformations. These differences can be explained by the following reasons:

(1) The different distances along the Z axis between the borophene and GaN/ZnO monolayers in van der Waals heterostructure supercells: the distance between the borophene and GaN was 2.91 Å, the distance between the borophene and ZnO was 2.51 Å. At shorter distances, the interaction between the layers in heterostructure will be more noticeable and, therefore, will lead to more noticeable changes in the properties of the heterostructure as compared to the properties of its constituent monolayers. This is also indicated by difference in the calculated binding energies for the heterostructure supercells. Due to the shorter distance between borophene and ZnO, the binding energy of the borophene/ZnO heterostructure (−0.80 meV) will be greater than the binding energy of the borophene/GaN heterostructure (−0.50 meV). In addition, a shorter distance between borophene and ZnO causes more noticeable changes in the atomic structure of the ZnO monolayer in the borophene/ZnO heterostructure as compared to changes in the atomic structure of the GaN monolayer in the borophene/ZnO heterostructure. In particular, the dihedral angles of the GaN and ZnO monolayers differ greatly: for GaN, it is negative and amounts to −7.21, while for ZnO, it is positive and amounts to 19.18.
(2) The differences in the features of the band structure of graphene-like GaN and graphene-like ZnO monolayers. The band structure of graphene-like GaN has an indirect band gap, while graphene-like ZnO has a direct band gap [38,39]. In addition, based on DFT calculations using GGA-PBE approximation, Xia et al. found that the VBM and CBM of graphene-like ZnO are located at the center of the Brillouin zone (Γ point), while the VBM of graphene-like GaN is located at the K point of the Brillouin zone [40]. The authors attribute this difference to the insufficient hybridization of the 4p-orbitals of Ga atoms and 2p-orbitals of the N atoms at the Γ point of the Brillouin zone, which follows from the lower PDOS peaks near the VBM for graphene-like GaN. Finally, according to both known calculated data and experimental data, the size of the band gap for graphene-like GaN and graphene-like ZnO also differs [41,42,43,44,45].

## 5. Conclusions

Thus, based on the first-principles calculations, the regularities of the influence of uniaxial and biaxial stretching/compressive deformations on the electronic structure of borophene/GaN and borophene/ZnO van der Waals heterostructures have been revealed. It has been found that the borophene/GaN heterostructure is more sensitive to axial compression, while the borophene/ZnO heterostructure is more sensitive to axial stretching. The differences are due to the different values of the distance between the 2D monolayers in borophene/GaN and borophene/ZnO heterostructures, as well as the features of the band structure of the GaN and ZnO monolayers, in particular, the different positions of VBM and CBM relative to the points of the Brillouin zone. For the appearance of an energy gap in the band structure of borophene/GaN and borophene/ZnO heterostructures, it is necessary that the zero DOS near the Fermi level be present in the PDOS of both borophene atoms and GaN/ZnO atoms. The p-orbitals of boron and nitrogen atoms make the decisive contribution to the band structure of the borophene/GaN heterostructure, and the p-orbitals of boron and oxygen atoms make the decisive contribution to the band structure of the borophene/ZnO heterostructure. It can be assumed that borophene/GaN and borophene/GaN heterostructures with electronic properties tuned by stretching/compression have prospects for an application as a material for nano- and optoelectronics, including devices with a Schottky barrier.
